# Intralymphatic Immunotherapy (ILIT) with both grass and birch allergen- a randomized controlled trial

**DOI:** 10.1186/2045-7022-5-S4-P29

**Published:** 2015-06-26

**Authors:** Laila Hellkvist, Eric Hjalmarsson, Susanna Georén Kumlien, Ola Winqvist, Ulla Petersson Westin, Lars Olaf Cardell

**Affiliations:** 1 Karolinska Institutet, Stockholm, Sweden; 2 Karolinska Institutet, Clinical Sciences, Intervention and Technology, Stockholm, Sweden; 3 Karolinska Institutet, Department of Medicine, Stockholm, Sweden; 4 Lund University, Skåne University Hospital, Malmö, Department of Otorhinolaryngology, Malmö, Sweden

## Background

Allergen specific immunotherapy is an effective treatment of allergic rhinitis. It is most commonly administered as repeated subcutaneous injections or as a daily sublingual tablet during 3-5 years. In order to shorten the treatment duration previous studies have evaluated the use of intralymphatic injections (ILIT) with promising results. This study assesses safety and efficacy of ILIT with two allergens given simultaneously.

## Method

60 patients 2012-2014 with moderate to severe birch and mild to moderate grass pollen allergy were recruited. They were randomized 1:1 to three ultrasound guided intralymphatic injections with placebo or ALK Alutard Birch and Grass 1000 SQ/U in each groin with one-month interval. The quality of life was assessed at peak pollen season using the rhinitis related quality of life (QoL) questionnaire Sinonasal Outcome test (SNOT-22). Allergen specific IgE and IgG4 were monitored and flow cytometry was used to characterize blood and lymph node aspirate.

## Results

36 patients have been analyzed so far (data from 2012-13). 14 patients received active treatment and 22 placebo. Baseline characteristics including disease severity and QoL were the same in both groups. All side effects reported were mild; 8% of active patients had local swelling at injection site. In the placebo group the QoL scores worsened during peak birch season (mean of difference between off and during season: 19.70±4.60, n=20, p= 0.0004). In contrast, the scores remained unchanged in the active group (6.27±4.72, n=11, p=0.21).S- Birch IgG4 levels increased 2 weeks after active ILIT (mean difference 0.26±0.11 mg/L, p=0.04, n= 11). No such change was seen after placebo (0 ±0.02 mg/L, p=0.97, n=17). S-Birch IgE levels increased 6-9 months after treatment in the active group (mean difference 12,05±4.58 kU/ml, p=0.02, n=12) but not in the placebo group (-0.05±5.1 kU/ml, p=0.99, n=21).Th1 cells (indicated as CCR5+ CD4+ cells) increased in blood after active ILIT (4.0±1.1%, p=0.04) but not after placebo (-0.6±0.7, p=0.44). These cells also tended to increase in the lymph node. Memory T-cells in blood also increased following active treatment (p=0.05).

## Conclusion

ILIT targeting birch and grass simultaneously appears to be a safe procedure. The treatment improves the quality of life during pollen season. This supports the idea of ILIT as an effective alternative route for specific immunotherapy.

